# Maternal and Fetal Outcomes of Pregnancy in Nephrotic Syndrome Due to Primary Glomerulonephritis

**DOI:** 10.3389/fmed.2020.563094

**Published:** 2020-12-10

**Authors:** Rossella Siligato, Guido Gembillo, Valeria Cernaro, Francesco Torre, Antonino Salvo, Roberta Granese, Domenico Santoro

**Affiliations:** ^1^Unit of Nephrology and Dialysis, Department of Clinical and Experimental Medicine, University of Messina, Messina, Italy; ^2^Department of Biomedical, Dental, Morphological and Functional Imaging Sciences, University of Messina, Messina, Italy; ^3^Unit of Gynecology, Department of Human Pathology of Adult and Childhood “G. Barresi”, University of Messina, Messina, Italy

**Keywords:** pregnancy, nephrotic syndrome, focal and segmental glomerulosclerosis, membranous nephropathy, membranoproliferative glomerulonephritis, minimal change disease

## Abstract

Chronic kidney disease (CKD) affects 3% of pregnancies, impacting on maternal and fetal outcomes, and at the same time, a recurrent question in nephrology regards gestation impact on kidney function. Observational studies stated that CKD stage, pre-existent hypertension, and proteinuria are the main predictors of possible complications, such as maternal CKD progression, maternal or fetal death, prematurity, small for gestational age (SGA) newborn, or admission to the neonatal intensive care unit. In this regard, given the prominence of proteinuria among other risk factors, we focused on primary nephrotic syndrome in pregnancy, which accounts for 0.028% of cases, and its impact on materno-fetal outcomes and kidney survival. Data extracted from literature are scattered because of the small cohorts investigated in each trial. However, they showed different outcomes for each glomerular disease, with membranous nephropathy (MN) having a better maternal and fetal prognosis than focal and segmental glomerulosclerosis (FSGS), membranoproliferative glomerulonephritis (MPGN), or minimal change disease (MCD). Nephrotic syndrome does not have to discourage women to undertake a pregnancy, but the correct management may include a specific evaluation of risk factors and follow-up for adverse materno-fetal events and/or maternal kidney disease progression.

## Introduction

In the past, fear of chronic kidney disease (CKD) progression could prevent nephrologists from sustaining their female patients' idea of conceiving or carrying on gestation, even if evidence of adverse outcomes was scattered. Today, mild CKD affects nearly 3% of women in childbearing age, whereas stage 3–5 CKD can be detected in 0.7% of same age women (1). However, the real prevalence in pregnant patients cannot be assessed, given that gestational screening guidelines usually include only urinalysis to identify urinary tract infections (UTIs) or proteinuria. On the other hand, mild CKD reports at more extended laboratory examinations performed in pregnancy are rising, likely because of increasing maternal age and higher incidence of hypertension, obesity, diabetes mellitus type 2 (DMT2), or smoking habits. Thus, first pregnancy is frequently the real touchstone to discover metabolic disorders, impaired renal function, or UTIs. Among primary kidney diseases, glomerulopathies, particularly nephrotic syndrome, are rare conditions whose management in pregnancy must include specific follow-up and evaluation of risk factors for adverse materno-fetal events and/or maternal renal disease progression. All the trials based on miscellaneous CKD cases demonstrate that stage of renal impairment, but above all baseline proteinuria and hypertension, are significant risk factors for adverse outcomes. The aim of our review is to investigate the outcomes of women affected by nephrotic syndrome due to primary glomerulopathies, including focal and segmental glomerulosclerosis (FSGS), minimal change disease (MCD), membranous nephropathy (MN), and membranoproliferative glomerulonephritis (MPGN).

## Nephrotic Syndrome and Pregnancy, A Difficult Data Analysis

Ethical concerns about pregnancy prevent starting interventional randomized controlled trials, so literature in the field is mainly based on retrospective or, to a lower extent, prospective observational studies. Moreover, statistical analysis of materno-fetal or kidney outcomes in those trials is often based on CKD stage rather than on the specific underlying causes that range from hypertension and/or proteinuria, diabetic nephropathy (DN), and glomerulonephritis to autosomal dominant polycystic kidney disease (ADPKD), kidney malformations, nephrolithiasis, or chronic UTIs. It is worth considering that some of them may expose patients to specific risks, such as elevated rate of fetal deformity in DN or hereditary transmission of ADPKD, in addition to other adverse outcomes as maternal CKD progression or maternal and fetal death, prematurity, small for gestational age (SGA) babies, or admission to the neonatal intensive care unit (NICU), which are common for all CKD causes. Finally, long-term effects of moderate–severe CKD have to be contemplated and result in a higher incidence of hypertension and ischemic heart disease in women affected by materno-fetal complications. Except for IgA nephropathy (IgAN) and systemic lupus erythematosus that result in the most common glomerular diseases in childbearing age women, investigating peculiar outcomes of other glomerulopathies appears to be quite complex because it is hard to find a pregnant cohort big enough to reach statistical significance after stratification of individual risk factors.

### Superimposed Preeclampsia or Kidney Disease Progression?

Nephrotic syndrome is estimated to occur in 0.028% of gestations (2), but an increased or new-onset proteinuria has to be distinguished from preeclampsia. Usually, time and clinical presentation are diagnostic criteria when proteinuria rises twice the baseline value if it is >2 g/day or 5-fold if it is lower, after gestation week 20, associated with the onset of hypertension and severe headaches or epigastric pain, as well as with laboratory abnormalities, such as thrombocytopenia and titer of serum aspartate aminotransferase >70 U/L (3).

At the moment, podocyturia and nephrinuria have been considered as direct urinary markers of podocyte damage. Podocyturia has higher sensitivity (88%) and specificity (79%) with immunofluorescence techniques targeting podocalyxin rather than synaptopodin or podocin. Nephrinuria is defined as an increase in urine nephrin level, preceding the onset of clinical manifestation of preeclampsia by about 9 days, with the best sensitivity (100%) and specificity (97%) during the 3rd trimester of pregnancy. However, their benefit as predictors of preeclampsia may not be extremely relevant in differential diagnosis between this condition and a new-onset or progression of nephrotic syndrome. Placental growth factor (PlGF) is an angiogenic factor whose production increases until gestation weeks 26–30 in healthy women. On the contrary, in preeclampsia, reduced titer of PlGF may be an expression of a placental dysfunction. Soluble FMS-like tyrosine kinase receptor 1 (sFlt-1) counteracts PlGF and also demonstrated to downregulate the expression of slit diaphragm components responsible for proteinuria. sFlt-1/PlGF ratio appears to be a promising placental specific marker of preeclampsia, in terms of both specificity and sensitivity (4), and correlates with disease severity, anticipating its onset by about 5 weeks.

### Specific Outcomes in Primary Nephrotic Syndrome

Maternal and fetal outcomes in FSGS patients were studied from 1980 to 2017 in several case series of pregnant women affected by biopsy-proven primary glomerular diseases. Except for a prospective trial and two retrospective studies each including only a case of FSGS and reporting, respectively, a case of progression of kidney disease, stillbirth, and preterm delivery (5–7), other studies included a number of pregnancies ranging from 10 to 84, with term delivery in 16–78.5% of cases (8–14). The analysis of adverse materno-fetal outcomes showed a high rate of incidence of spontaneous abortion (5.9–28.6%), preterm delivery (5.9–58.8%), and perinatal death (11.7–45.2%) (8–14). Regarding maternal renal outcomes, hypertension emerged in 20–71% of pregnancies, but persisted after 6 months after delivery in 4–66% of women (8–14). The highest rate of postpartum hypertension was reached in a Saudi cohort of 84 pregnancies in 18 women affected by FSGS, who had undergone 38 pregnancies before the onset of nephrotic syndrome. Malik et al. compared the materno-fetal outcomes of their pregnancies demonstrating no statistical significance of the increased abortion rate registered after FSGS diagnosis. Conversely, higher rate of preterm delivery (5.9%, *p* = 0.0006) and SGA infants (16.6%, *p* = 0.0001) in FSGS cohort than in control group gestations resulted to be significant (13). Interestingly, proteinuria increased from 3.8 ± 3.4 g/day in the first pregnancy to 4.8 ± 2.0 g/day in the last one, but the difference was not significant (*p* = 0.275). At last follow-up, occurring at a mean of 10.5 ± 3.3 years after FSGS diagnosis, half of their cohort had CKD progression, and four women (22.2%) reached end-stage renal disease (ESRD), requiring renal replacement therapy (13). These results are in line with the previous experience of Packham et al., who reported a transient impairment of renal function in 49% of pregnancies, persisting in four patients and leading to ESRD in three of them (19%), respectively, after 2, 7, and 18 years, in contrast to higher renal survival rate (93% at 10 years from diagnosis) of a control group of 58 non-pregnant women affected by FSGS (11).

Six studies evaluated outcomes of pregnancy and MCD (2, 5, 6, 8, 14). Two retrospective studies, including only seven MCD patients in their report, showed 50–66% of SGA newborns. Regarding renal outcomes, they appeared limited to gestation, with increased level of proteinuria and onset of hypertension registered in only a pregnancy for each trial, both resolving postpartum (2, 5). In line with these results, Surian (8) recorded no complications in two pregnancies. In 1985, Abe et al. studied 17 pregnancies of 10 MCD patients, highlighting materno-fetal complications (SGA infants, preterm delivery, polyhydramnios, premature abruptio placentae, and severe bleeding after delivery) in 12% of gestations and fetal loss and abortion in 12 and 17% of cases, respectively. The main risk factors identified were hypertension and glomerular filtration rate <70 ml/min (6). More recently, a retrospective trial included seven pregnancies of which 71.4% hesitated in preterm delivery and 14.3% in fetal loss beyond gestation week 20. Only a patient experienced a stable increase in proteinuria postpartum (14).

Trials investigating MN and pregnancy highlighted a reduced impact of this glomerulopathy on the incidence of abortion and stillbirths in the absence of risk factors, such as nephrotic range proteinuria during pregnancy (2, 5, 6, 8, 9, 12, 14–20). A transient development of hypertension was registered in 7.7–45.4% of pregnancies, and increased proteinuria in up to 60% of cases confirmed an association of MN with worse fetal and maternal outcomes. Packham et al. reported the highest rate of materno-fetal complications with fetal loss in 24% of cases, preterm delivery in 43% of pregnancies, CKD progression in 9%, but the onset of hypertension in 46% of pregnant patients, and higher proteinuria in 54.5% (17). A Saudi cohort study comparing the maternal outcomes of nine women experiencing pregnancies before (*n* = 21) and after (*n* = 30) MN diagnosis demonstrated that the different rates of abortion (4.7 vs. 3.3%, *p* = 0.843), preterm delivery (0 vs. 6.6%, *p* = 0.152), and perinatal mortality (0 vs. 3.3%, *p* = 0.317) between the two groups were not statistically significant (18). Moreover, the rise in mean serum creatinine level experienced from the first gestation after MN diagnosis to the last one of the same cohort was also not significant (mean SCr 68 ± 17 vs. 80 ± 48 μmol/L, respectively, *p* = 0.489) (18).

MPGN defines a pathologic pattern characterized by subendothelial, subepithelial, and mesangial deposits of immune complexes (due to infections, autoimmune or monoclonal disorders) or of complement (due to alternative pathway dysregulation). Idiopathic MPGN cases were studied in five trials accounting for a total number of 36 patients (5–9). Two studies included only a patient and obtained opposite results, with a neonatal death (6) and a physiologic pregnancy (7). The other retrospective trials showed full-term deliveries in up to 50% of patients, whereas 16.7–23.8% had preterm delivery. Fetal death was more frequent in FSGS and MN patients evaluated in the same studies, with both an abortion rate and peripartum mortality in 0–11.1% of gestations (5–9). Moreover, Barcelò et al., stratified MPGN, FSGS, and MN patients' outcomes based on proteinuria and detected a significant association only of proteinuria levels >2.5 g/day with preterm delivery and low birth weight (1,930 ± 301 g) (9).

New data come from De Castro et al.'s retrospective study on 26 pregnancies in 19 patients affected by biopsy-proven primary nephrotic syndrome with a mean proteinuria of 8.33 ± 6.7 g/day (19). In 26 pregnancies described, glomerulopathies most represented were FSGS (42%), MN (16%), and IgAN (16%), followed by MCD, MPGN, C3 glomerulonephritis (C3GN), and fibrillary glomerulonephritis, each accounting for 5%. A new diagnosis was performed in 12 women during pregnancy at mean gestational week 18.6 ± 9.1, and kidney biopsy was performed in eight patients within a mean gestational age of 21 weeks. Only two minor biopsy complications were reported in this group of women (hematoma and hematuria), compared with one adverse event recorded in 11 patients who had pathologic diagnosis before or after their pregnancies (1 small arteriovenous fistula), but the procedure proved to be worth the risk because it allowed to change the management of nephrotic syndrome in six patients who started prednisone in a dose ranging from 60 to 120 mg/day. One patient affected by known C3GN was given eculizumab. The main adverse maternal outcomes described were preeclampsia (27%), acute kidney injury (AKI, 23%), and cellulitis (12%), which was not associated with steroid therapy. FSGS accounted for half of AKI episodes, one of which progressed to ESRD, whereas the remaining happened in IgAN and C3GN patients. Fetal outcomes included preterm delivery and low birth weight (under 2,500 g) in 54% of pregnancies, intrauterine growth restriction (IUGR) in 11%, and NICU admission in 31%. Two cases of premature rupture of membranes were identified in patients treated with high dose glucocorticoids. In this cohort, MN confirmed having the best maternal–fetal and kidney survival outcomes, with three patients with stable serum creatinine during pregnancies and a transient increase in proteinuria, which reverted to non-nephrotic range in a year after delivery. Statistical analysis of risk factors of the whole cohort confirmed the strong association of proteinuria with adverse outcomes, such as preeclampsia (*p* = 0.002), low birth weight (*p* = 0.002), and preterm delivery (*p* = 0.02) (19).

The most recent retrospective study performed by Liu et al. reported preserved kidney function but the occurrence of adverse materno-fetal complications including fetal loss (11%), preterm delivery (26%), and severe preeclampsia (15%) in 27 pregnancies from 2008 to 2018 in a cohort of women affected by MN (20). Consistent with De Castro' observations (19), Liu also demonstrated a correlation of proteinuria before week 20 (intended as both time-averaged urine protein, OR 67.5, *p* < 0.001; maximum proteinuria >3.5 g/day, OR 29.3, *p* = 0.001), hypoalbuminemia (time-averaged albumin, OR 67.5, *p* < 0.001; minimum serum albumin <25 g/L, OR 10.9, *p* = 0.01), and no-remission during pregnancy (OR 21.6, *p* = 0.004) with adverse materno-fetal outcomes. In particular, time-averaged proteinuria and serum albumin were associated with birth weight percentile of neonates. Moreover, anti-PLA2R antibody positivity (*p* = 0.03) appeared as a specific risk factor for pregnant MN patients (20). Interestingly, a case report of PLA2R positive MN patient undergoing pregnancy demonstrated the transplacental passage of these autoantibodies, which were detected in the fetus' cord blood (21). Sachdeva et al. confirmed this finding in a new case report and also showed a PLA2R passage to newborn during breastfeeding, which raises questions about the opportunity of this practice and of checking PLA2R titer, serum albumin, and urinalysis in children of mother affected by primary MN (22).

### Symptomatic Treatment of Nephrotic Syndrome in Pregnancy

Primary glomerulonephritis may require the administration of immunosuppressive drugs, such as mycophenolic acid and cyclophosphamide, whose teratogen risk is known and should therefore be replaced before gestation (23). Monoclonal antibodies, such as anti-CD20 rituximab, should be avoided because of their transplacental transport in the last trimesters of pregnancy, which can induce transient B cell depletion in fetuses (24). Conversely, short half-life corticosteroids, such as prednisone and methylprednisolone, are considered a valid alternative because of their inactivation by placental 11β-hydroxysteroid dehydrogenase type 2 that prevents steroid adverse effects on fetus (23). Calcineurin inhibitors (CNIs), such as cyclosporin (CsA) or tacrolimus (TAC), appear to be safe, with no evidence of increased risk of teratogenicity (25), and experimental rat models show how CsA transplacental passage is impaired by P-glycoprotein (26).

Angiotensin-converting enzyme inhibitors (ACEIs) and/or angiotensin II receptor blockers (ARBs) are widely recommended in KDIGO guidelines as the first-line therapy of nephrotic syndrome for their nephroprotective effects (27). Their teratogen effect on the cardiovascular, urinary, skeletal, and central nervous systems makes them contraindicated in pregnancy particularly during the second trimester of gestation, so general advice is to have them immediately discontinued at the beginning of pregnancy (28–30). Hoeltzenbein et al. conducted a prospective observational trial comparing 329 women accidentally exposed to ACEIs during their first trimester of gestation and no longer than week 20 and 654 control pregnant women without a history of hypertension (31). The significantly increased rate of major birth defects in the ACEIs cohort confirmed the results of previous studies on these drugs in pregnancy (32, 33), but they also investigated maternal hypertension impact on malformations, already demonstrated in other trials (34, 35). Comparing patients affected by chronic hypertension exposed to ACEIs vs. patients treated with methyldopa, they concluded that malformations rate was not significantly different, also suggesting the potential role of severe hypertension *per se* as a risk factor for adverse fetal outcomes (31).

A safe option to maintain blood pressure in pregnant women affected by CKD within values of 135/85 mmHg is represented by α2 adrenergic receptor blocker, such as methyldopa, whereas clonidine may impact fetal growth, and it is usually avoided for high risk of hypertensive rebound at discontinuation. Other antihypertensive choices are represented by calcium blockers, such as long-acting nifedipine or labetalol, a combined α- and β-blocker, with efficacy and safety comparable to methyldopa. Other β-blockers represent a second choice because of the risk of adverse events in newborns (usually transient bradycardia, hypotension, and hypoglycemia). Diuretics are usually avoided in pregnancy and may be adopted in nephrotic syndrome refractory to immunosuppressive therapy under strict control to avoid volume depletion and AKI risk.

Regarding albumin infusion in nephrotic syndrome in pregnancy, no study is conclusive about its potential benefit. On the contrary, hyperfiltration risk has to be considered, with a paradox increase in proteinuria, and this discourages the systematic use of albumin in this setting (36, 37).

Nephrotic syndrome is also characterized by a hypercoagulability state that has to be treated when the albumin level is lower than 2 g/dl. Low molecular-weight heparin is a valid choice for both prophylaxis and treatment of thromboembolic complications during pregnancy and postpartum, because it does not cross the placenta. Heparin should be discontinued before labor induction or planned cesarean section, but can be resumed after delivery (38). During pregnancy, low-dose acetylsalicylate is also a useful option preventing preeclampsia in high-risk patients, such as CKD and proteinuric patients. Despite guidelines suggesting to start therapy after the first trimester to avoid hemorrhagic risk in case of spontaneous abortions, the protective effects on placentation are higher when acetylsalicylate is started early (39, 40).

The positive effect of a low protein diet with supplementation of amino acids and ketoacids in pregnant CKD patients has to be considered as a therapeutic option, in the absence of a specific anti-proteinuric therapy (41, 42).

## Conclusions

Our review focused on materno-fetal and kidney survival outcomes reported in pregnant patients with nephrotic syndrome (Table 1). Baseline levels of proteinuria, as well as hypertension and CKD stage, are the most important risk factors for poor outcomes, as confirmed in other trials involving pregnant women affected by CKD. Incidence of C-sections and preterm deliveries are higher than healthy controls, as reported by Piccoli et al.'s findings in a prospective trial in CKD patients (54.8 and 33.4%, respectively) (43). Among nephrotic syndromes, MN resulted to be the most favorable in terms of outcomes, whereas MPGN and FSGS expose patients to a higher degree of both materno-fetal and renal adverse events. Adequate counseling appears to be indispensable to increase women awareness of the benefit of pregnancy planning to manage potential teratogen therapies and to minimize risks of gestational complications and disease progression, especially if a complete and stable remission is not obtained before conceiving. More prospective trials are required to better evaluate specific predictors of severe adverse outcomes and to reach statistical significance after stratifying individual risk factors.

**Table 1:** Retrospective trials on primary nephrotic syndrome and materno-fetal and renal outcomes.

	**Pregnancies/patients**	**Abortions**	**Preterm delivery**	**SGA**	**Neonatal death**	Hypertension during gestation/follow-up^*^	Proteinuria during gestation/follow-up^*^
**FSGS**							
Katz et al. (5)	1/1	0	0	0	0	0	1 (100)/0
Surian et al. (8)	25/19	2 (8)	6 (24)	1 (4)	4 (16)	5 (20)/1 (4)	0/0
Abe et al. (6)	1/1	0	0	0	1 (100)	1	1 (100)/1 (100)
Barcelò et al. (9)	17/13	1 (5.9)	3 (17.6)	0	0	4 (23.5)/1 (7.7)	3 (17.6)/1 (7.7)
Kincaid-Smith and Fairley (10)	28/15	8 (28.6)	14 (50)	0	0	20 (71)/3 (18)	11 (39)/4 (14)
Packham et al. (11)	31/21	0	12 (38.7)	5 (16.1)	14 (45.2)	23 (68)/4 (12.9)	15 (49)/4 (19)
Jungers et al. (12)	10/6	1 (10%)	3 (30)	3 (30)	1 (10)	0/0	4 (40)/1 (16.6)
Alexopoulos et al. (7)	1/1	0	1 (100)	0	0	1 (100)/0	1 (100)/1 (100)
Malik et al. (13)	84/18	7 (8.3)	5 (5.9)	14 (16.6)	11 (13)	52 (61)/12 (66)	13 (72)/12 (71)
O'Shaughnessy et al. (14)	17/10	0	10 (58.8)	4 (23.5)	2 (11.7)	11 (68.8)	5 (38.5)/3 (37.5)
De Castro et al. (19)	13	0	2	2	0	0	5 (38.5)/5 (38.5)
**MCD**							
Studd and Blainey (2)	4/4	0	0	2 (50)	0	1 (25)	0/0
Katz et al. (5)	6/3	0	1 (16.6)	4 (66.6)	0	1 (16.6)/0	1 (16.6%)/0
Surian et al. (8)	2/2	0	0	0	0	0/0	0/0
Abe et al. (6)	17/10	3 (17.6)	2 (11.8)	2 (11.8)	2 (11.8)	1 (5.9)/0	1 (5.9)/0
O'Shaughnessy et al. (14)	7	0	5 (71.4)	0	1 (14.3)^*^	0/0	2 (28.6)/1 (14.3)
De Castro et al. (19)	1	0	0	0	0	0	1 (100)/1 (100)
**MN**							
Studd and Blainey (2)	12/7	2 (6.6)	4 (33.3)	11 (41.6)	0	8/0	0/0
Forland and Spargo (15)	5/4	2 (40)	0	0	0	0/0	3 (60)/3 (75)
Noel et al. (16)	9/7	1 (11.1)	0	0	1 (11.1)	0/0	1 (11.1)/1
Katz et al. (5)	10/7	0	1 (10)	1 (10)	0	4 (40)/5 (71.4)	6 (60)/4 (57.1)
Surian et al. (8)	8/7	0	0	0	0	1 (12.8)/0	0/0
Abe et al. (6)	13/7	0	1 (7.7)	0	1 (7.7)	1 (7.7)/0	0/0
Barcelò et al. (9)	9/7	0	2 (22.2)	0	0	3 (33.9)/0	2 (22.2)/0
Packham et al. (11)	33/24	8 (24)	14 (43)	11 (33)		15 (46)/0	2 (22.2)/0
Jungers et al. (12)	37/17	13 (35)	(30%)	0	0	(35)/0	0/0
Malik et al. (18)	30/9	1 (3.3)	2 (6.6)	3 (10)	1 (3.3)	1/1 (11.1)	0/0
O'Shaughnessy et al. (14)	6	0	2 (33.3)	0	1 (16.6)^*†*^	3 (75.0)	2 (33.3)/1 (16.6)
De Castro et al. (19)	4	0	1 (25)	1 (25)	0	0/0	4 (100)/0
Liu et al. (20)	27/25	0	7 (25.9)	7 (25.9)	1 (3.7)	0/0	0/0
**MPGN**							
Katz et al. (5)	4/4	0	0	1 (25)	0	3 (75)/1 (25)	4 (100)/3 (75)
Abe et al. (6)	1/1	0	0	0	1 (100)	1 (100)/0	0/1 (100)
Surian et al. (8)	18/14	2 (11.1)	3 (16.6)	2 (11.1)	2 (11.1)	4 (22.2)/3 (21.4)	3 (16.6)/2 (14.3)
Barcelò et al. (9)	21/16	2 (9.52)	5 (23.8)	0	1 (4.76)	5 (23.8)/2 (12.5)	4 (19)/2 (12.5)
Alexopoulos et al. (7)	1/1	0	0	0	0	0	0

*SGA, small for gestational age; FSGS, focal and segmental glomerulosclerosis; MCD, minimal change disease; MN, membranous nephropathy; MPGN, membranoproliferative glomerulonephritis*.

* *Renal outcomes during follow-up have been evaluated in percentage on the number of patients in each trial and not on pregnancies total number*.

† *Fetal death after 20 weeks of gestation. All values represent n (%)*.

## Author Contributions

RS, GG, and DS: conceptualization and writing—original draft preparation. RS, VC, GG, and DS: methodology. RS and DS: investigation. RG and DS: resources. RS, RG, and VC: data curation. RS, RG, AS, FT, VC, and DS: writing—review and editing. RS, RG, AS, FT, and DS: visualization. RG, DS, and GG: supervision. All authors contributed to the article and approved the submitted version.

## Conflict of Interest

The authors declare that the research was conducted in the absence of any commercial or financial relationships that could be construed as a potential conflict of interest.
